# Disorders of gastrointestinal hypomotility

**DOI:** 10.12688/f1000research.8658.1

**Published:** 2016-08-01

**Authors:** Klaus Bielefeldt, Ashok Tuteja, Salman Nusrat

**Affiliations:** 1 Department of Internal Medicine, University of Pittsburgh Medical Center, Pittsburgh, PA, USA; 2 Department of Medicine, University of Utah, Salt Lake City, UT, USA; 3 Department of Medicine, Oklahoma University Medical Center, Oklahoma City, OK, USA

**Keywords:** Gastrointestinal hypomotility, diagnostics, gut motor function, brain-gut axis, visceral activity, hypomotility disorders

## Abstract

Ingestion and digestion of food as well as expulsion of residual material from our gastrointestinal tract requires normal propulsive, i.e. motor, function. Hypomotility refers to inherited or acquired changes that come with decreased contractile forces or slower transit. It not only often causes symptoms but also may compromise nutritional status or lead to other complications. While severe forms, such as pseudo-obstruction or ileus, may have a tremendous functional impact, the less severe forms of hypomotility may well be more relevant, as they contribute to common disorders, such as functional dyspepsia, gastroparesis, chronic constipation, and irritable bowel syndrome (IBS). Clinical testing can identify changes in contractile activity, defined by lower amplitudes or abnormal patterns, and the related effects on transit. However, such biomarkers show a limited correlation with overall symptom severity as experienced by patients. Similarly, targeting hypomotility with pharmacological interventions often alters gut motor function but does not consistently improve symptoms. Novel diagnostic approaches may change this apparent paradox and enable us to obtain more comprehensive information by integrating data on electrical activity, mechanical forces, patterns, wall stiffness, and motions with information of the flow of luminal contents. New drugs with more selective effects or more specific delivery may improve benefits and limit adverse effects. Lastly, the complex regulation of gastrointestinal motility involves the brain-gut axis as a reciprocal pathway for afferent and efferent signaling. Considering the role of visceral input in emotion and the effects of emotion on visceral activity, understanding and managing hypomotility disorders requires an integrative approach based on the mind-body continuum or biopsychosocial model of diseases.

## Introduction

Normal gastrointestinal (GI) function requires a system capable of adjusting to, at times, rapidly or dramatically shifting volumes due to food intake, fragmentation of larger ingested particles, and mixing and movement of chyme to bring nutrients to the absorptive sites and ultimately to expel residual materials from the gut. Many of these tasks depend on forces generated by the smooth muscle cells found in the mammalian gut. Abnormalities of GI motility, whether inherited, acquired, or induced by medications, may thus have significant implications on nutrient intake, transport, absorption, and fecal output. For this review, we will focus on one aspect of motor dysfunction: hypomotility. We will try to define underlying mechanisms, the consequences on the mammalian gut, and our ability to diagnose and treat it as a potential cause for disease. We will primarily use decreased contractile force as our operational definition of hypomotility, relate it to transit whenever possible, and focus on clinical aspects rather than the molecular or physiological mechanisms.

The mammalian, and thus also the human, gut has a basic structural organization that includes distinct muscle layers. The innermost muscularis mucosae separates the mucosa from the submucosa and likely contributes to the movement of chyme in the microenvironment close to the absorptive surfaces ^1^. However, relatively little is known about its role in human disease. The more prominent and better-studied muscularis externa layer contains fibers oriented in circular and longitudinal directions that form its inner and outer component, respectively. For the purpose of this review, we will discuss hypomotility largely based on the assumption that contractile forces and patterns generated by this external layer play a key role in the tasks outlined above ^2^.

## Mechanisms of hypomotility

Considering the role of muscle activity, disorders of smooth muscle function, such as inherited abnormalities of contractile proteins, by definition contribute to the development of hypomotility ^3, 4^. Recent studies suggest that other molecular defects may lead to subtle, but potentially more common, manifestations. For example, detailed molecular and physiologic investigations identified changes in a voltage-sensitive sodium channel in patients with irritable bowel syndrome (IBS) ^5, 6^. In addition to inherited abnormalities or susceptibility, patients may also acquire changes involving contractile proteins, ion channels, or other molecules, as has been shown for diabetic gastroparesis ^7, 8^.

Muscle cells ultimately generate forces and create the motor events we can observe, but they are regulated by several cell types, which may be responsible for disorders characterized by hypomotility. The interstitial cells of Cajal form a network of functionally coupled cells within the muscle layer of the GI tract, where they generate and transmit electrical activity that controls smooth muscle function ^9^. Animals with congenital absence of these cells do not display normal electrical activity and have significant abnormalities of motility and transit ^10^. Consistent with experimental data, inherited and acquired, potentially reversible changes have been identified in various motility disorders ^10– 12^. Recent studies raise questions about a potential role of macrophages as modulators of GI motility. These macrophages form a three-dimensional network within the muscle layers and produce a variety of mediators that can alter gut function ^13, 14^.

Innervation plays an important role in the regulation of GI motility. The intrinsic or enteric nervous system forms the myenteric plexus with ganglia being located between the circular and longitudinal portions of the muscular layer. Localized abnormalities, such as inherited aganglionosis of the rectum (Hirschsprung’s disease) or acquired loss of ganglion cells in achalasia, disrupt the normal pattern of activity and, in the context of these disorders of sphincteric structures, delay or even block the passage of luminal contents ^15, 16^. Changes in enteric neurons or their function may also contribute to a variety of disorders characterized by abnormal motility, such as esophageal dysfunction in Sjögren’s syndrome, gastroparesis, pseudo-obstruction, or chronic constipation ^11, 17– 22^. Extrinsic innervation provides a link between the central nervous system and the GI tract, often referred to as the brain-gut axis. Based on anatomic and functional criteria, extrinsic innervation is typically divided into sympathetic and parasympathetic components, with the vagus nerve being the predominant component of the parasympathetic branch of the autonomic nervous system. Experimental approaches have defined its important modulatory influences in many different areas, which range from motility and secretion to immune and endocrine function. The clinical impact can be seen in patients who have undergone a surgical vagotomy as a treatment of ulcer disease, which often leads to gastric atony, impaired opening of the pyloric channel, and prolonged retention of ingested material ^23^. Vagotomies are rarely performed nowadays, but unintentional vagal injury during foregut surgery or autonomic neuropathy may contribute to the development of motility disorders, such as dyspeptic symptoms after anti-reflux surgery or diabetic gastroparesis ^24– 26^.

Inherited or acquired connective tissue disorders may manifest with impaired GI motility. The network of connective tissue provides the scaffolding for muscle cells. Few mechanistic studies systematically examined the exact physiological role of this passive support, yet the consequences of systemic sclerosis or inherited disorders, such as Marfan or Ehlers Danlos syndrome, clearly highlight its importance ^27– 29^. Other problems, such as vascular or joint manifestations, often predominate the classic manifestations of these rare diseases. However, less severe phenotypes, such as joint hypermobility syndrome, are more common and – as shown recently – seem to also be associated with a high prevalence of functional GI disorders ^30, 31^.

By far the most important, but not always fully appreciated, cause for changes in GI motility is the use of medications. A host of different agents can decrease contractile forces, change patterns of contractions, and/or delay transit of material throughout the gut. The list includes agents that are even available over the counter, such as anti-histamines or loperamide, but also prescription medications, such as opioids, calcium channel blockers, nitrates, anticholinergic substances, several antidepressants, antipsychotics, or anti-emetics, to mention just a few of the more common culprits.

## Consequences of hypomotility

Considering the important role of normal GI motility in assuring entry into and transit through the gut with mixing and fragmentation also facilitating absorption, hypomotility should compromise normal GI function and nutritional status and/or cause symptoms. Consistent with these theoretical considerations and the previously mentioned role of medications as a cause of motor dysfunction, blunting contractile amplitudes through antimuscarinics, opioids, and L-type calcium channel blockers delays orocecal transit and alters meal-induced changes in colonic activity, likely contributing to the development of constipation that is often seen with these agents ^32– 36^. Conversely, cholinergic agonists increase contractile forces and improve esophageal clearance of swallowed fluids ^37, 38^. Independent of such pharmacologic investigations as proof of concept, detailed studies of esophageal motility clearly show the relevance of normal contractile function. A complete lack of normal peristaltic activity as an extreme case of hypomotility is very rare in asymptomatic individuals and is typically associated with dysphagia ^39^. More limited changes with localized decrease in contractile amplitudes below 30 mmHg correlate with impaired bolus clearance if they involve a sufficiently long segment of the esophagus ^40, 41^ but may not necessarily trigger significant symptoms ^42– 45^.

This correlation between low contractile amplitudes as an operational definition of hypomotility and impaired transit or symptoms is even less consistent when we look at gastric function. While antimuscarinic agents slow gastric emptying in healthy volunteers ^46^, the L-type calcium channel blockers nifedipine and verapamil have no effect ^47, 48^. Similarly, manipulation of transit with erythromycin or morphine in healthy persons does not correlate with consistent and significant changes in contractile indices measured with a wireless capsule ^49^. Conversely, activation of cholinergic pathways with bethanechol or neostigmine increases contractile activity but does not accelerate gastric emptying ^46, 50^. Perhaps most importantly, neither emptying nor an aggregate measure of contractile activity, the motility index (MI), correlates consistently with symptoms, which may in part be due to limited coordination of contractions within functional units of the GI tract ^51– 56^. Increased activity triggered by a meal or pharmacologic stimulation is associated with higher ratings of dyspeptic symptoms ^50, 51, 57^, pointing at the role of more complex motor patterns rather than measures of force generation only. Detailed physiological testing supports such a conclusion, as abnormal patterns are common in patients with severe GI dysfunction or functional dyspepsia ^58– 60^ and correlate with altered transit of ingested material through the GI tract ^61, 62^. While we have a more complex understanding of the many different factors controlling GI motility, the clinical manifestations and results of diagnostic testing still largely fit into the dichotomous concept of the previously proposed neurogenic or myogenic mechanism of dysmotility that separates abnormalities in patterns from those defined by abnormal amplitudes ^63^.

## Assessment of hypomotility

**Figure 1.**
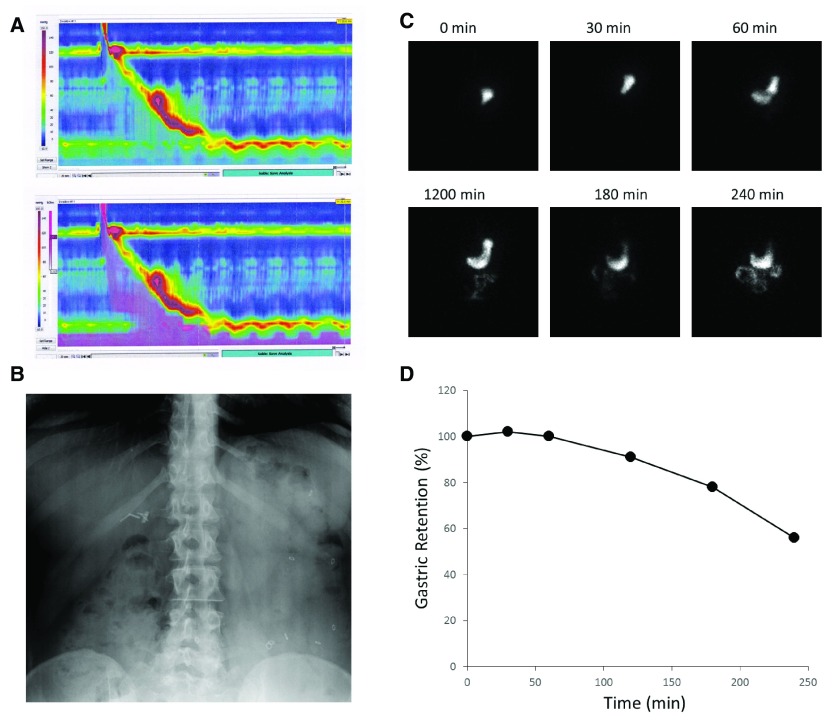
Examples of clinically used assessments of gastrointestinal (GI) motility. Panel **A** demonstrates a pseudocolor display of esophageal pressure changes in response to a swallow (upper panel). The associated changes in impedance, caused by the traversing fluid bolus, are superimposed in purple in the lower panel. In Panel **B**, radio-opaque markers can be seen in the stool-filled colon (mostly accumulated on the left side) and allow an estimate of whole gut transit time. Gastric emptying of a radioactively labeled meal is documented with intermittent scintigraphic imaging (Panel **C**) and plotted as a function of time (Panel **D**).

The very principles of functional GI testing were introduced more than 100 years ago, when gastric intubation with rubber tubes allowed the measurement of residual contents after a test meal, the first recordings of pressure changes, and – with the advent of radiography – the indirect visualization of contractions and emptying ^64^. While the techniques have been refined and advanced significantly, they still focus on direct or indirect recording of contractions and the resulting movements of luminal contents ( Figure 1). The direct assessment of contractile forces typically employs catheter-based systems and is thus invasive. Esophageal and anorectal manometry have become routine diagnostic tools in modern medicine; antroduodenal and colonic manometry require more complex instrumentation and prolonged recordings and have unclear diagnostic utility, which have limited their application in clinical practice. The miniaturization and wireless signal transfer brought us a capsule-based system that measures pH and pressure as the capsule is propelled through the gut. The pH allows us to approximate its location (the stomach is highly acidic, and fermentation lowers the pH to about 5 in the proximal colon). This technique has opened up options to non-invasively assess motor activity and entry/exit rather than transit *per se* ^65, 66^. Yet it provides limited information about motility patterns, which would require prolonged recordings from multiple sites in stable and predefined locations.

Focusing on the movement of luminal content rather than contractions offers an alternative endpoint for clinical and scientific investigations of GI motility. The most commonly employed approaches largely rely on radioactive molecules that label physiologically relevant substrates that can be followed with scintigraphic methods ( Figure 1C&D) ^67, 68^. For slower phenomena, such as the determination of whole gut transit, intermittent X-rays suffice to determine the number and location of retained radio-opaque markers ( Figure 1B), which allows us to calculate an approximate transit time ^69, 70^. While not used as often, we can exploit changes in the microenvironment of different compartments within the GI tract to determine transit times. Early studies relied on the urinary excretion of mostly colored labels that were absorbed after reaching the small bowel. As this approach has urine production and bladder emptying as confounders, the temporal resolution is quite limited. Nowadays, the same principle typically uses substrates that are absorbed or fermented, diffuse across membranes, and ultimately reach the lungs where they are exhaled and can be easily captured in ‘real time’ to assess gastric emptying or orocecal transit ^71– 73^.

**Figure 2.**
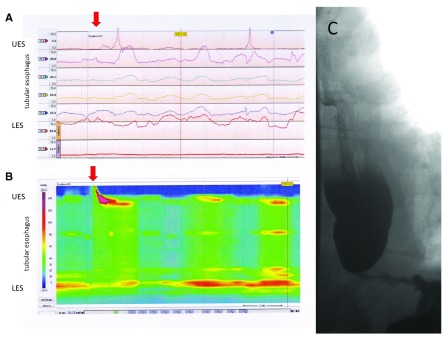
Different test results and their representation obtained in a patient with the esophageal motility disorder achalasia. Pressure recordings obtained at different levels can be displayed as line tracings, showing the typical manometric results in this disorder with aperistalsis in the tubular esophagus and incomplete relaxations of the lower esophageal sphincter (LES) (Panel **A**). The same findings are shown as high-resolution esophageal pressure topography, with the results of many different recording sites being color-coded and with a seamless display of the entire esophageal length based on real and extrapolated data (Panel **B**). The corresponding contrast study (Panel **C**) shows a dilated esophagus with contrast retention and smooth tapering of the distal esophagus with a non-opening LES.

Conceptually, contractile activity and patterns will ultimately propel ingested material along the axis of the GI tract. Thus, both endpoints should relate to each other as shown for achalasia as an example in Figure 2. However, direct assessments suggest an, at times, poor correlation between overall test results of manometry and transit studies ^74^. This led to the development of approaches that assess both parameters in parallel. The combination of pressure measurements and impedance changes after the ingestion of typically a liquid, but at times viscous bolus, has now become a routine test in clinical practice ( Figure 1A) ^43, 75^. Simple constraints due to more difficult access, more complex motor pattern, and the need for longer recording times require a different strategy for the assessment of gastric, small bowel, or colonic contractions and transit measurements. While these strategies are not yet ready for routine application, investigators combined assessments of luminal filling and wall motions of segments within the GI tract using computerized reconstructions of cross-sectional imaging, such as magnetic resonance imaging (MRI). In the colon, dynamic MRI shows a good correspondence between high amplitude propagating pressure waves assessed manometrically and luminal diameter changes with fluid propulsion, triggered by intracolonic infusion of the laxative bisacodyl ^76^. While many reports still refer to amplitudes of observed contractions, this analysis is based on wall motion or diameter changes with amplitudes being defined by geometric rather than pressure differences ^77– 79^. Parallel assessment at several points over time may enable the investigator to recognize patterns that can then be correlated with movement of luminal contents. Adding specifically designed magnetic markers may even allow us to directly measure transit or velocity of movement within the gut lumen ^80^. While this is labor intensive and costly, initial results show promise in differentiating disease from health, separating between different disorders ^79, 81^, and identifying the effect of pharmacological interventions on contractile activity and movement of luminal content ^78, 82^. Thus, we have a proof of concept demonstrating that assessment of complex structure-function relationships is possible and may provide more insight into disease mechanisms or treatment options.

## Treatment of hypomotility

During the last two decades, physicians and scientists have tried to translate our better understanding of GI motor function into better treatment for GI motor dysfunction. While we did indeed make progress, a retrospective viewpoint gives the process the appearance of a rollercoaster ride. The discovery of erythromycin’s effects as a potent motilin agonist gave physicians a prokinetic with effects on gastric and small bowel motility ^83, 84^. However, the unwanted antibiotic effects, the potential for adverse effects, and drug-drug interactions as well as an apparent loss of efficacy due to tachyphylaxis led to the development of several alternatives. Before looking at some of these alternatives, clinicians experimented with another macrolide, azithromycin, which had similar effects when studied acutely but comes with fewer drug-drug interactions than erythromycin. Despite this conceptual advantage, evaluations do not allow conclusions, as they were limited to short-term assessments with manometry ^85, 86^. More importantly, there are still the antibiotic properties as a major drawback. With the encouraging initial data, motilin agonists have surfaced and resurfaced since, with ABT-229 ^87^, KC 11458 ^88^, and mitemcinal all improving symptoms but not demonstrating true benefits when compared to placebo ^89, 90^. After a brief hiatus, another agent, camicinal, has been shown to accelerate gastric emptying in healthy volunteers without affecting esophageal, small bowel, or colonic transit ^91^. While the jury is still out on the utility of this most recent agent, new observations may rekindle interest in motilin agonists. We typically use these agents to stimulate gastric motility and emptying, yet studies in healthy volunteers point at another potential use. When asked to rate symptoms during recordings of normal gastric motor activity, participants reported higher hunger scores during phase III of the cyclical activity pattern, seen in the fasting state ^92^. This period of clustered and propagating activity fronts is associated with an increase in motilin, prompting follow-up experiments with motilin agonists. Interestingly, these agonists triggered clustered contractile fronts that propagated distally and heightened hunger feelings, which was not seen after the administration of a cholinergic agonist that simply increased the frequency and amplitude of contractions without a phase III-like activity. Conversely, the investigators also noted that unexplained anorexia is associated with loss of phase III ^93^. With nutritional problems, most importantly the obesity epidemic we are facing, such observations may translate into novel applications in the future.

An extensive body of research has established the role of serotonin (5-HT) with its many different receptor subtypes in regulating gut function, motivating clinicians and drug companies to explore the therapeutic potential of serotoninergic agents. The initial results were promising. Cisapride, with its mixed agonistic and antagonistic properties, enhanced contractile forces and accelerated transit, even though symptomatic benefit was less consistent ^94– 96^. The agent was eventually withdrawn from the market, as interactions with an inwardly rectifying potassium channel prolonged the cardiac repolarization phase and led to torsade de pointes ^97^. The convincing basic science and the observed effects of cisapride prompted the development of several other agents with more specific binding to the 5-HT _4_ receptor. Activation of this pathway facilitates acetylcholine release, which modifies intrinsic signaling and could potentially improve motor function and maintain physiologically relevant patterns ^98^. While conceptually appealing and backed by strong preclinical studies, the track record of these agents is littered with problems. Alosetron, which did not target hypomotility but slowed down transit and was approved as a selective 5-HT _3_ antagonist for the management of diarrhea-predominant IBS, was withdrawn owing to an increase in cases of ischemic colitis ^99, 100^. Tegaserod, the first and only selective 5-HT _4_ agonist approved in the US, accelerated transit ^101^ and improved constipation in women with or without IBS symptoms but was linked to an unexpected rise in myocardial infarction, leading to its withdrawal from the market ^102^. Interestingly, while studies and marketing emphasized the benefit on pain and discomfort in IBS ^103, 104^, the only comparative effectiveness analysis studies did not show superiority over a simple osmotic laxative with a better risk profile ^105^. Within the last few years, several newer agents have been tested in preclinical and clinical studies ^106– 114^. Concerns about side effects ^115, 116^, limited efficacy ^117– 122^, loss of efficacy over time ^123^, and equivalence or even inferiority compared to cheaper and safer agents ^124– 127^ have continued to raise questions about the cost-benefit ratio and true utility of this class of agents. Given that abnormalities in 5-HT levels and release are linked to IBS, perhaps more direct targeting of 5-HT release is worthwhile.

Cholinergic agents have been available for a long time, with atropine and hyoscyamine being part of the 'pharmacopoeia' of physicians for centuries. Considering the role of acetylcholine in neuromuscular transmission, it makes intuitive sense to use agonists as a treatment for hypomotility. For acute colonic pseudo-obstruction, enhancing cholinergic signaling has indeed become the first-line therapy ^128^. In the esophagus, contractile amplitudes increase ^37, 38^. However, despite these acute effects on esophageal physiology, there was no tangible benefit in terms of reflux symptoms or acid exposure ^129^. Studies on dysphagia are largely restricted to the management of myasthenia gravis, which demonstrate a benefit of cholinesterase inhibitors but obviously target neuromuscular transmission of skeletal muscle affected in this disorder. Similarly, gastric contractions increased after administration of cholinergic agonists ^46, 130^, yet emptying did not improve and symptoms may even worsen ^46^. In diabetic patients, the cholinesterase inhibitor pyridostigmine similarly did not alter gastric emptying but showed a benefit in patients with chronic constipation ^131, 132^. Thus, the picture is at best mixed and highlights that patterns rather than simple contractile forces generated play an important role in normal gut function.

In the last decade, observations related to the peptide hormone ghrelin have generated quite a bit of interest about its possible utility in patients with impaired gastric function or dyspepsia. Levels rise prior to food intake, regulate appetite, and modulate gastric emptying ^133, 134^. Initial experiments were encouraging, as they showed that infusion of ghrelin or short-term use of ghrelin agonists improved dyspeptic symptoms and gastric emptying ^135– 137^. Effects seemed to go beyond the regulation of gastric motility, as the ghrelin agonist relamorelin increased meal-induced propagating contractions and accelerated colonic transit in small trials of patients with constipation ^138, 139^. Another ghrelin agonist, ulimorelin (TZP 101), shortened the time to first bowel movement after partial colectomy in an initial dose-finding study ^140^ but was not superior to placebo in the larger trials on postoperative ileus ^141^. Initial studies of this agent in gastroparesis were similarly promising ^137, 142, 143^. However, further development of its oral analogue has been halted after larger trials did not show benefit over placebo in gastroparesis ^144, 145^. Dyspeptic symptoms, such as nausea, and impaired gastric emptying have been linked to changes in electrical activity that can be recorded from the human stomach ^60, 146, 147^. These observations led to the idea that implantation of stimulation electrodes may entrain the basic electrical rhythm, thereby indirectly improving coordination of contractile activity, emptying, and ultimately dyspeptic symptoms. Initial experiments with temporarily implanted electrodes demonstrated the feasibility in humans and motivated the development of systems that could be permanently implanted for gastric electrical stimulation (‘gastric pacers’) ^148– 150^. Subsequent experiments optimized the stimulation parameters, which now employ a high frequency that does not target gastric muscles and motility, is not associated with consistent changes in emptying, and presumably works through modulation of afferent input ^151^. While open label studies suggested significant benefit, controlled trials did not support superiority over sham stimulation ^152^.

## Questions and directions for future research

Using increasingly sophisticated techniques, we can now assess contractile forces, patterns, and transit in the various functionally distinct compartments of the GI tract. However, we are still lacking reliable diagnostic and predictive markers. In patients with dyspeptic symptoms, delays in gastric emptying and their treatment-associated changes do not correlate with symptom improvement ^153^. Considering the complex responses to food intake, we may need to shift from a focus on facilitating emptying and consider other mechanisms, such as impaired accommodation ^154^. A more nuanced assessment of symptoms or mechanisms may enable us to target a subset of symptoms, as, for example, for postprandial bloating, which tends to respond better to prokinetics ^155, 156^. While antroduodenal or colonic manometry may predict treatment responses in subsets of patients ^157, 158^, the true utility of these assessments has never been examined systematically, with conclusions being largely based on small cohort studies of skewed patient groups. As described in this article, we have focused on physiologic variables when approaching patients with symptoms and possible gut dysfunction. However, recent studies of functional illnesses defined by altered motility or transit show the impact of psychological factors as confounders, which may not only influence the perceived symptom severity but also drive healthcare-seeking behavior ^159– 163^ or predict treatment outcomes ^164^. Considering the conceptual importance of brain-gut interactions, future studies need to define to what extent these correlations are consequences of altered physiology or whether the GI manifestations are primarily somatic manifestations of emotional or other psychiatric problems.

Several decades after the introduction of functional testing into clinical practice, we still have limited options to assess small bowel and colonic function. The wireless motility capsule may well point in the right direction, but it still provides too little insight, considering the importance of motility patterns discussed above. Nanoparticles, perhaps combined with specifically designed adhesive gels, may allow non-invasive monitoring of motor activity, patterns, and relevant changes in the local microenvironment ^165^. Such approaches may prove useful beyond diagnostic strategies, as they could be combined with focused/localized drug delivery.

The last few years have clearly moved our attention to a previously often-neglected component of the luminal contents: microbial colonization. Several interesting studies have emerged and should attract our attention. Perhaps not surprisingly, changes in nutrient-microbiome interactions altered GI transit in a mouse model, which is in part driven by poorly absorbed materials and thus confounded by fermentation and likely increases in the volume of colonic contents ^166, 167^. However, volume changes explain only a part of this complex interplay, as specific microbial species and their metabolites modulate serotonin content in enteroendocrine cells, which in turn affect gut function ^168, 169^. While probiotics have long been a part of medical management, their effects are still limited ^170^, showing the need for a better understanding of the triad of food, gut, and microbes.

Electrical stimulation found its way into the medical management of GI disorders more than a century ago ^64^. The results of gastric electrical stimulation described above show continued shortcomings. Despite ongoing problems, similar approaches were recently tried in constipation. Early open label studies reported promising results ^171^ but also raised concerns about long-term efficacy and safety with high rates of problems and reoperations, which have been shown in sacral neurostimulation for other indications ^172^.

GI motility matters. It allows us to ingest and digest food and ultimately expel the residue. Normal motor function relies on the complex interplay of the central and peripheral nervous system, different cell types in the GI muscle wall, and the luminal contents. Decreasing forces or altering patterns of normal contractions, the correlates of ‘hypomotility’, can interfere with transit, lead to symptoms, and/or compromise nutritional status. New development may enable us to better measure and analyze contractile forces, patterns, and transit, use them more effectively as biomarkers of hypomotility, identify new targets for our interventions, and understand the complex relationship that emerges from the brain-gut axis and closely links emotion with gut function and symptoms that ultimately determine quality of life.
